# Gastrotomy for Intestinal Obstruction

**Published:** 1853-05

**Authors:** 


*Gastrotomy for Intestinal Obstruction.* (Under the care of Mr. HANCOCK in Charing-cross Hospital.)—It occurs, unhappily but too often, that cases of unconquerable obstruction of the bowels are met with in our hospitals; and in casting a retrospective glance at our series of nosocomial reports, we find that we have had to record several cases of this kind. Another instance of intestinal obstruction has just taken place at Charing-cross Hospital, and we hasten to lay before our readers the particulars of the case.

Ann H——, aged 52, single, following the occupation of cook, and residing at Ham Common, was admitted on the 11th of February, 1853, under the care of Mr. Hancock. The patient had enjoyed very good health until ten months before admission, when she was suddenly seized with a severe pain in the bowels, which latter became much distended, and remained constipated for five days. She was attended by Dr. Hassall, and took various purgative medicines, without effect, up to the fifth day, when an enema was administered through a long tube (which the patient stated was passed some distance into the bowel), and the same evening a motion was passed. After this she appears to have improved, but has ever since been obliged to take aperients frequently to keep the bowels open, and has observed that the motions have been getting smaller—that is, long and thin, being at last about the size of her little finger.

About a month before admission, the patient began to suffer much from distension of the bowels, and considerable flatulence, which at times was so inconvenient that she was obliged to lie upon her bed until the flatus passed off. This state of things lasted for about a week, the bowels being at the same time rather constipated. Three weeks before the woman came to the hospital, she became so unwell that she was obliged to give up work and go to bed; and during the whole of that day and night she had violent vomiting and retching. An injection was administered during the day, and a motion passed. Enemata were given several times during the following week, with the same effect, the motions consisting of small scybala, the last one having been passed fifteen days before admission. At that time the motions, according to the patient's statement, had assumed the form of pills. Frequent vomiting took place up to the time of admission, when it ceased, and from this time it did not occur after food, but generally after taking any aperient medicine. The matter brought up was yellow, brown or greenish, and had a bitter, sourish taste, the smell not being particularly disagreeable.

The bowels had been much distended, and they were greatly so on admission. At times there was difficulty in passing urine, the latter coming away in small quantities. The patient did not suffer much pain except from borborygmi, which were relieved as soon as the flatus escaped, which at this period always occurred upwards, excepting after an injection, when a little passed downwards. No pain in any particular spot was complained of, excepting from a little distension about the umbilicus. The patient stated that the enemata did not appear to go beyond a certain point—a little above the crest of the left ilium; and she thought that nearly a quart of injection has been retained at one time. She was bled the night before admission, and had calomel and opium. Mr. Hancock ordered two grains of calomel and a quarter of a grain of opium to be taken every second hour.

Second day: Pulse 108, rather small, but not particularly feeble; tongue furred, brown in the centre and red at the top and sides; surface warm; no perspiration; countenance not at all anxious. The spirits are pretty good, and the woman is tolerably cheerful. She does not sleep well; appetite very bad; abdomen tympanitic, supple and painless. There is rather a fæcal smell about the patient. Third day: pulse weak, 112; does not feel so well as yesterday, and is very low; is in great pain with flatus. She vomited a small quantity of yellow bitter matter this morning. Tongue more furred; skin cool and dry; urine passed freely.

A consultation was held at two o'clock, and it was decided to give an enema, and meet again at four o'clock. The enema was administered through the tube of a stomach pump, which was introduced in its whole length into the bowel, and about a quart of injection used, as no greater quantity could be employed. It returned, however, almost directly, untinged by fæcal matter, but accompanied by a small quantity of flatus. The temperature of the ward was raised as much as possible, and warmed sheets and towels provided, to cover the patient, should the operation be performed.

Fourth day, four P. M.: Nothing having passed from the bowels, and it being now the seventeenth day since anything had done so, the operation was decided upon, and chloroform administered. As there were not sufficient indications as to the situation of the obstruction, Mr. Hancock determined to commence by an incision in the median line from the umbilicus to the pubis; the intestines, distended by flatus, escaped through this opening, and were immediately covered by warmed towels, to preserve their temperature. The transverse colon being distended, the cause of obstruction was sought beyond, and found, without any difficulty, at the sigmoid flexure, in a portion of the bowel about an inch in length, constricted by a band about half an inch wide, but of so long standing as to have thickened the intestine and obliterated its canal. This band was divided, but the gut was so changed in structure and compressed, that it was evident the only chance of recovery consisted in opening the colon, and forming an artificial anus above the obstructed point. A transverse incision was therefore carried through the abdominal parietes, from below the umbilicus to the crest of the ilium on the left ssde, and an opening being made in the colon about an inch in length, the cut edges of the gut were attached by sutures to the margins of the integumentary wound; after which the intestines were returned into the cavity of the abdomen, the wound brought together by sutures, and the patient sent to her bed.

The patient bore the operation very well, her pulse remaining pretty good throughout, and being 126 at its termination. Stimulants were administered two or three times during the operation, which lasted forty minutes from first to last; and a little brandy, with fifteen minims of laudanum, immediately after. Twelve P. M.: Pulse stronger, 126; surface of body cold; a large quantity of fæcal matter has passed through the opening; the patient complains greatly of pain in the part incised. Mr. Hancock saw her at nine o'clock, and as she then appeared extremely low, ordered her laudanum and ammonia, in camphor mixture, every four hours. The pain, which was most severe, now became less intense.

First day after the operation, eight A. M.: Mr. Hancock found the patient better; her pulse was fuller; skin warm, and covered with warm perspiration; bowels have acted very copiously during the night through the artificial opening, and the vomiting has ceased; pain has diminished, and the woman has had some sleep. Ten A. M.: not quite so well; pulse more feeble; in other respects much the same. Three P. M.: Much worse; is in great pain; countenance anxious; very restless; upper and lower extremities cold; pupils contracted; tongue dry and parched; pulse cannot be counted; can bear pressure without much increase of suffering. The patient died at a quarter past three o'clock.

No regular post-mortem examination took place, but it was easily ascertained that the obstruction lay principally in the locality which has been mentioned above.—*Lond. Medical Circular.*

